# Guest Hospital, Dudley

**Published:** 1918-08-24

**Authors:** Arthur Bird

**Affiliations:** Secretary. Guest Hospital, Dudley


### THE EDITOR'S LETTER-BOX.

### GUEST HOSPITAL, DUDLEY.

*To the Editor of* The Hospital.

Sir,—The article in The Hospital headed "The Day of Reckoning at the Guest Hospital, Dudley," has been brought to the notice of my Board, and I am directed to ask for your permission to submit to you the following facts.

The article in the *Dudley Herald* of July 6, on which your article is based, is entirely inconsistent with the laudatory articles and reports as to the work and efficiency of the hospital which have from time to time appeared in the same organ, and the change of tone is apparently inspired by personal rancour rather than by interest in the welfare of the hospital.

Our beds number 115, and generally our wards are full. Frequently the number of beds occupied is over 100. The admissions average 1,350 a year. If the number of beds had been reduced to fifty, or reduced at all, the Board would agree with you that there was something radically wrong with the management, but fortunately there is no need and no intention to make any reduction.

During recent years about £15,000 has been spent in improvement, extensions, and new buildings. These include the addition of eight beds to the women's ward, with two small wards of three beds each; a new operating theatre for women; a new children's ward, light and airy, with sixteen cots, and verandahs on two sides for open-air treatment. New lavatories and bathrooms have been thrown out from the men's wards, and an entirely new system of drainage, for the whole of the hospital, put in. The laundry has been doubled in size and equipped with new machinery.

The hot-water supply is excellent in every part of the hospital, provided by two large new boilers. Only two years ago, on land given by the Earl of Dudley, a big new surgical out-patient department and eye infirmary was built, and this hospital was one of the first in the Midlands to adopt the Local Government scheme for the treatment of venereal diseases, and treats a very great number of cases.

These particulars, which are indisputable facts, should convince you that not only is there no standing still or closing of beds, but that the policy of the committee is to go forward and benefit the quarter of a million people in this district to the fullest extent possible. It seems a pity that some inquiry was not made before accepting as true the malicious statements of a local paper, and it is hoped that you will make the *amende honorable* by counteracting the wrong and false impression given.—I am, Sir, your obedient servant,

Arthur Bird, Secretary.

Guest Hospital, Dudley.
August 12, 1918.

[The statement in the *Dudley Herald* that the number of beds at the Guest Hospital, Dudley, had been cut down to fifty is one that we are glad to have authoritatively contradicted, but, so far as we are aware, it was not denied until it was reproduced in The Hospital. The interesting developments detailed by Mr. Bird do not touch the criticisms directed against the mode of administration, the method of appointment complained of, or the failure of the recent appeal. And till they have been disposed of, the "policy to go forward," like the success of the appeal, will be indefinitely delayed.—Ed. The Hospital.]

### THE INSURANCE ACT AND ITS ADMINISTRATORS.

*To the Editor of* The Hospital.

